# NFX1-123 is required for keratinocyte differentiation and HPV 16 DNA maintenance in early and persistent infection models

**DOI:** 10.1128/jvi.00649-26

**Published:** 2026-06-15

**Authors:** Maura A. Dankoski, DeShawn Thompson, Kevin M. Quist, Rachel A. Katzenellenbogen

**Affiliations:** 1 Translational Cancer Biology Program, IU Simon Comprehensive Cancer Center, Indiana University School of Medicine 12250, Indianapolis, Indiana, USA; 2 Department of Pediatrics, Indiana University School of Medicine 12250, Indianapolis, Indiana, USA; Tufts University, Medford, Massachusetts, USA

**Keywords:** differentiation, infection, NFX1-123, HPV

## Abstract

Persistent high-risk human papillomavirus (HR HPV) infections cause nearly 5% of cancers worldwide, including cervical and oropharyngeal cancers. HR HPVs sustain long-term infections in stratified epithelial tissues by maintaining copies of viral genomes in basal keratinocytes and utilizing the cellular differentiation processes in the upper layers to coordinate key steps in the viral life cycle. We have demonstrated previously that the endogenous host protein NFX1-123 bound directly to the HR HPV type 16 oncoprotein E6 (16E6), and that this partnership increased keratinocyte proliferation and differentiation, two processes that are both required for persistent HPV 16 infections. As there is a range of endogenous expression of NFX1-123 in normal basal keratinocytes, we aimed to investigate the role of NFX1-123 abundance on the initiation and persistence of HPV 16 infections. In this study, we utilized keratinocytes with overexpressed, endogenous, or reduced levels of NFX1-123 and infected these cells with an HPV 16 quasivirus (Qsv) to interrogate changes to keratinocyte growth and viral DNA copy numbers with NFX1-123 modulation. Keratinocytes with reduced NFX1-123 had blunted differentiation and stratification in raft cultures, and this correlated with decreased HPV 16 Qsv DNA copies. Similarly, when we reduced NFX1-123 in W12E cells, a cervical cell line harboring episomal HPV 16 genomes, we saw a decrease in raft culture differentiation and thickness, and fewer viral genome copies compared to W12E cells with endogenous NFX1-123. These findings indicate the importance of NFX1-123 on the initiation and maintenance of HPV 16 infections that may persist and progress to cancer.

**IMPORTANCE:**

A high-risk human papillomavirus (HPV) infection that persists for decades is the biggest risk factor for the development of cervical and other HPV-associated cancers. The ability of HPV to maintain its genome and persist in stratified epithelium is dependent on its partnership with host proteins and its co-opting of cellular pathways. Here, we investigate how the abundance of HPV 16’s host protein partner NFX1-123 affects viral genome maintenance and replication in monolayer and three-dimensional cultures, modeling both early and persistent infection. This study reveals that NFX1-123 is required for keratinocyte differentiation and stratification in raft cultures, and the disruption of these processes significantly impedes the ability of HPV 16 to maintain its episomal genome at consistent copy numbers. These findings indicate the importance of NFX1-123 for the persistence of HPV 16 infections and encourage further investigation of NFX1-123 as a potential therapeutic target in HPV 16-positive cancers and precancers.

## INTRODUCTION

Human papillomaviruses (HPVs) are the most common sexually transmitted infection globally (1). More than 200 HPV genotypes have been identified to date, and these are categorized as either low risk or high risk based on their association with cancer development (2). High-risk HPVs are associated with virtually all cases of cervical cancers, as well as other anogenital and oropharyngeal cancers (2). Despite effective preventive and early interventions, such as prophylactic HPV vaccinations and screening strategies, respectively, cervical cancer remains the most common gynecologic malignancy worldwide (3). The greatest risk factor for the development of cervical cancer and other HPV-associated malignancies is a persistent, long-term infection with a high-risk HPV (4). Thus, extensive research has been conducted to uncover the conditions that promote persistent productive and oncogenic HPV infections.

High-risk HPVs initiate infection in basal keratinocytes of stratified squamous epithelium (5, 6). Upon infection, the viral genome is transported to the nucleus of the host cell as extrachromosomal double-stranded DNA (7). These infected basal cells form a reservoir for persistent HPV infection where HPV genomes are maintained at a constant copy number (5). As host keratinocytes begin to differentiate and migrate toward the epithelial surface, HPV utilizes this differentiation process to induce expression of viral late genes and undergo rapid amplification of its genome to thousands of episomal copies per cell (7). Assembly of viral progeny occurs in the terminally differentiating layers of stratified squamous epithelium, and infectious virus is released as host epithelial cells are sloughed off (5).

To facilitate viral genome replication and evade host immunosurveillance programs, HPVs utilize viral proteins that manipulate the host cell environment to promote viral persistence in basal keratinocytes and viral DNA amplification in terminally differentiated keratinocytes (5). The high-risk HPV type 16 (HPV 16) has two proteins widely considered to be oncoproteins, E6 and E7. HPV 16 E6 and E7 promote productive viral infection by perturbing apoptosis and promoting cell cycle activation through degradation of tumor suppressors p53 and Rb, respectively (6). The viral gene E2 can act as a transcription factor and regulates the expression of E6 and E7 throughout the viral life cycle to promote simultaneous cellular division and differentiation and to ensure viral immune evasion (6). These viral factors manipulate host cells to support the HPV life cycle, which can secondarily support oncogenesis if a high-risk HPV infection persists.

One potential consequence of persistent HPV infections is the integration of viral DNA into host chromatin over time; however, in productive HPV infections, the viral DNA is maintained as an extrachromosomal episome in the nucleus of the host cell throughout both early and late stages of the viral life cycle. Integration of HPV genomes into host DNA is thought to be spontaneous and sporadic, as integration events are a dead-end for the viral life cycle (5, 6, 8). Furthermore, integration breakpoints in the HPV 16 genome are usually located in the E2 gene, which disrupts E2-mediated repression of E6 and E7 expression (5). E6 and E7 levels are thus elevated in cells with integrated HPV DNA, resulting in increased host cell pathway dysregulation and contributing further to carcinogenesis.

Importantly, E6 and E7 do not themselves have enzymatic activity and must partner with host cell proteins to create an environment beneficial to the virus and to support oncogenesis. HPV 16E6 has been shown to partner with multiple cellular proteins to manipulate several cellular processes and create an environment beneficial to the virus (6, 9, 10). One host cell protein bound directly by 16E6 is NFX1-123, an endogenous cytoplasmic protein that has both RNA- and protein-binding functions (11–13). 16E6 hijacks the functions of NFX1-123 to post-transcriptionally regulate cellular growth, immortalization, and differentiation genes in keratinocytes (14). There is a range of endogenous NFX1-123 expression seen in basal keratinocytes of cervical stratified squamous epithelium, and this expression is typically increased in upper, differentiated keratinocytes (15, 16). A recent study found that 16E6 increased expression of the deubiquitinase USP9X, and this led to reduced ubiquitination and elevated total protein levels of NFX1-123 when 16E6 is expressed (17). In primary human foreskin keratinocytes (HFKs) expressing 16E6, increased expression of NFX1-123 has been shown to enhance cellular growth and longevity while promoting keratinocyte differentiation through post-transcriptional gene regulation (11, 15, 16, 18–20), processes which are normally mutually exclusive. Additionally, 16E6 and NFX1-123 collaboratively mislocalize innate immune signaling proteins, blunting the ability of keratinocytes to activate antiviral responses (21). With these findings, high NFX1-123 expression with 16E6 co-expression may promote a milieu beneficial to the HPV life cycle.

Although it has been shown that there is a range of endogenous NFX1-123 expression in normal cervical epithelium (15, 16), the role of baseline NFX1-123 abundance on the initiation and persistence of HPV 16 infections has not been previously studied. Here, we aimed to investigate how the expression of NFX1-123 affected the initiation and maintenance of HPV 16 infections. While NFX1-123 modulation did not appear to impact the infectivity of HPV, higher NFX1-123 abundance did confer a growth and differentiation advantage and supported increased viral copies in monolayer and raft cultures of infected HFKs, and this was lost with knockout of NFX1-123 in HFKs as well as in W12E cells. These studies reveal the importance of NFX1-123 to drive the growth of infected keratinocytes and the maintenance of productive HPV 16 infections that promote cancer development.

## RESULTS

### mCherry pseudovirus and HPV 16-Hygro quasivirus infected primary HFKs with a range of NFX1-123 expression

**Fig 1:**
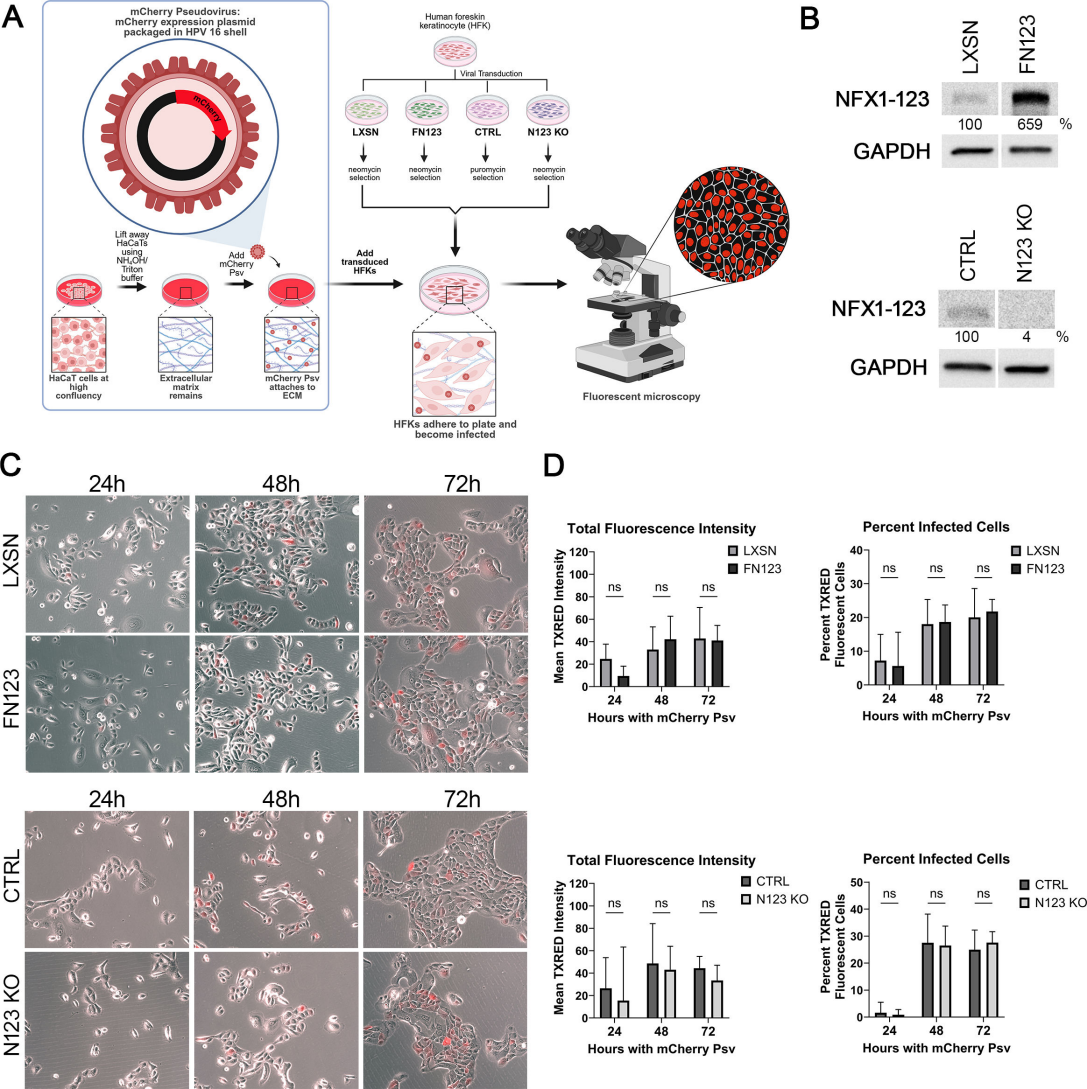
mCherry pseudovirus-infected HFKs with overexpressed, endogenous, or knocked out NFX1-123. (**A**) HFK cell lines were transduced with FLAG-tagged NFX1-123 (FN123), an LXSN control (LXSN), CRISPR-Cas9-mediated NFX1-123 knockout (N123 KO), or a CRISPR-Cas9 plasmid resulting in endogenous NFX1-123 (CTRL). Cells were then infected using a HaCaT-generated ECM with mCherry pseudovirus packaged in the HPV 16 capsid, and cells were imaged using fluorescent microscopy beginning 24 h after infection. Figure created with BioRender.com. (**B**) Immunoblotting of whole-cell protein extracts confirmed greater NFX1-123 in FN123 HFKs and reduced NFX1-123 in N123 KO HFKs. Densitometry of proteins (% expression) normalized to GAPDH and to LXSN or CTRL expression is listed. (**C**) FN123 and LXSN HFKs or N123 KO and CTRL HFKs were infected with mCherry Psv and imaged at 10× magnification using TXRED/*TRANS* overlay fluorescent settings 24, 48, and 72 h after infection. (**D**) Infection efficiency of HFKs with mCherry Psv was quantified by total red fluorescence intensity and percent infected cells. Error bars represent the 95% confidence intervals of five independent fields of view. One representative experiment from HFK A is shown from a total of four conducted in biologically independent HFK cell lines. ns, non significant.

Sporadic basal keratinocytes in the cervical epithelium show greater-than-typical expression of NFX1-123 (16). To investigate whether the abundance of NFX1-123 has an impact on the initiation of HPV 16 infections, we modeled this variation in expression in four biologically unique primary HFKs (HFK A, B, C, and D) that were transduced with either a FLAG-tagged NFX1-123 overexpression construct (FN123) or its matched control vector (LXSN) or a CRISPR-Cas9-mediated NFX1-123 knockout vector (N123 KO) or its matched control vector (CTRL) (Fig. 1A). Selection for these transduced cell populations was completed, and we confirmed the modulation of NFX1-123 protein levels by western blot (Fig. 1B). To investigate whether baseline NFX1-123 levels impacted the ability of the HPV 16 capsid proteins to successfully infect HFKs, we infected cells with an mCherry pseudovirus (Psv), which consists of an mCherry expression plasmid encapsidated in the HPV 16 shell (Fig. 1A). Cells were infected with mCherry Psv using an extracellular matrix (ECM) infection method, mimicking the natural steps of HPV infections (22) (Fig. 1A). Briefly, to generate the ECM, HaCaT cells were plated at high confluency before being removed, leaving the ECM undisturbed. mCherry Psv were then added and allowed to attach to the ECM before HFKs were added. Cells were imaged at 24, 48, and 72 h after mCherry Psv infection, and total red fluorescence intensity and percentage of red fluorescent cells were calculated at each time point (Fig. 1C through D). While increases in total fluorescence intensity and percentages of infected cells were observed over time, there were no differences in mCherry Psv infection associated with increased or decreased NFX1-123 in HFKs.

**Fig 2:**
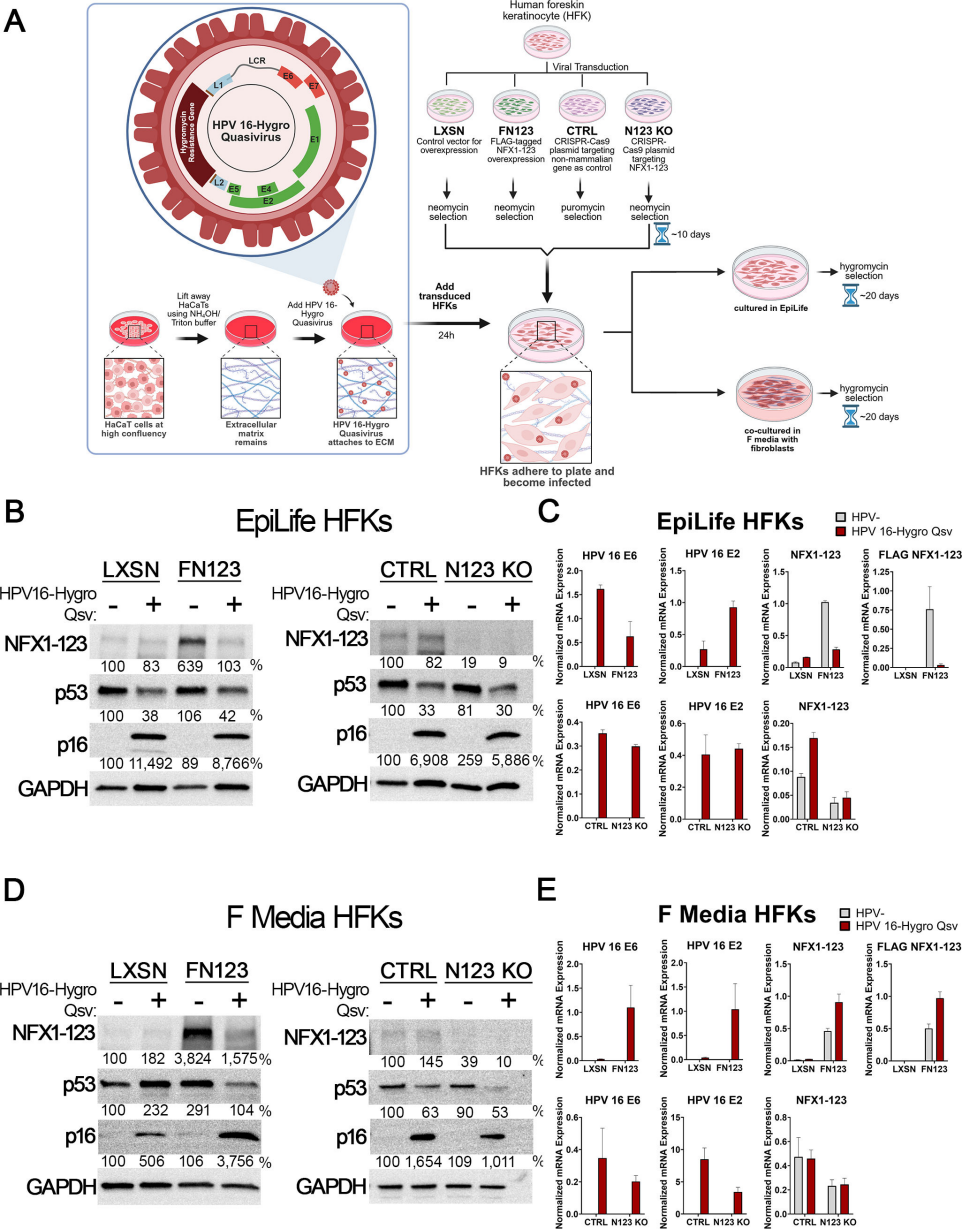
Validation of HPV 16-Hygro quasivirus infection in HFK cell lines with overexpressed, endogenous, and knocked out NFX1-123. (**A**) Experiment scheme for quasivirus infection. HFK cell lines were transduced with FLAG-tagged NFX1-123 (FN123), an LXSN control (LXSN), CRISPR-Cas9-mediated NFX1-123 knockout (N123 KO), or a CRISPR-Cas9 plasmid resulting in endogenous NFX1-123 (CTRL). Cells were then infected using a HaCaT-generated ECM with HPV 16-Hygro Qsv, which consists of the HPV 16 genome containing truncated late genes and a hygromycin resistance cassette packaged in the HPV 16 capsid. After 48–72 h of infection with HPV 16-Hygro Qsv, HFKs were split to fresh plates in either monoculturing conditions in serum-free (EpiLife) media, or in co-culturing conditions with mouse fibroblast feeder cells in serum-containing F media. In both conditions, HPV 16-Hygro Qsv-infected cells were selected for using 5–7.5 µg/mL hygromycin for approximately 20 days, at which point cells were collected for experiments. Figure created with Biorender.com. (**B**) Immunoblotting of FN123, LXSN, N123 KO, and CTRL HFKs infected with HPV 16-Hygro Qsv cultured in EpiLife confirmed greater NFX1-123 in FN123 cell lines, reduced expression of NFX1-123 in N123 KO lines, as well as reduced p53 and increased p16 levels in HPV 16-Hygro Qsv-infected cell lines. Densitometry of proteins (% expression) normalized to GAPDH and to uninfected LXSN or CTRL expression is listed. (**C**) HPV 16E6, HPV 16E2, NFX1-123, and FLAG-tagged NFX1-123 mRNA expression were measured in HFKs selected in EpiLife by RT-qPCR and normalized to 36B4. (**D**) Immunoblotting of FN123, LXSN, N123 KO, and CTRL HFKs infected with HPV 16-Hygro Qsv cultured in F Media confirmed greater NFX1-123 in FN123 cell lines, reduced expression of NFX1-123 in N123 KO lines, as well as reduced p53 and increased p16 levels in HPV 16-Hygro Qsv-infected cell lines. Densitometry of proteins (% expression) normalized to GAPDH and to uninfected LXSN or CTRL expression is listed. (**E**) HPV 16E6, HPV 16E2, NFX1-123, and FLAG-tagged NFX1-123 mRNA expression were measured in HFKs selected in F Media by RT-qPCR and normalized to 36B4. Representative experiments from HFK A are shown. Error bars represent the 95% confidence intervals of three technical replicates.

With confirmation that HFKs could be consistently infected using Psv, we repeated these ECM infection method experiments with HPV 16-Hygro Qsv (Fig. 2A). Cells infected with HPV 16-Hygro Qsv were selected for using hygromycin in two different monolayer conditions: monoculture in EpiLife media, which contains minimal growth factors and no sera; and co-cultured using the Rheinwald-Green method, which consists of co-culturing keratinocytes with irradiated mouse fibroblast feeders in a serum-containing media to encourage episomal maintenance of HPV 16-Hygro Qsv genomes after infection (23–25) (Fig. 2A). We confirmed overexpression of FLAG-tagged NFX1-123, CRISPR-Cas9-mediated knockout of NFX1-123, and HPV 16-Hygro Qsv infection at both mRNA and protein levels after serial transduction, infection, and selection for the presence of Qsv in both EpiLife and F Media culturing conditions. FLAG-tagged NFX1-123 mRNA was detected in the FN123 HFKs in both culturing conditions (Fig. 2C and E), and total NFX1-123 protein and mRNA were also increased (Fig. 2B through E), indicating the presence of additional NFX1-123. Knockout of NFX1-123 was confirmed by reduced expression of NFX1-123 mRNA and protein in the N123 KO HFKs (Fig. 2B through E). Endogenous NFX1-123 protein and mRNA were detected in HFKs containing each control vector (LXSN and CTRL) (Fig. 2B through E). HPV 16-Hygro Qsv was confirmed in LXSN, FN123, CTRL, and N123 KO HFKs cultured under both conditions by detecting HPV16 E6 and E2 mRNA (Fig. 2C and E). HPV 16 E6 and E7 protein activity were seen by the degradation of p53 and expression of p16 in infected HFKs (Fig. 2B and D). Although the overexpression of NFX1-123 protein in FN123 cells infected with HPV 16-Hygro Qsv was reduced compared to the uninfected FN123 cells, FN123 HFKs maintained increased NFX1-123 protein and mRNA compared to Qsv-infected LXSN cells when both were infected with HPV 16-Hygro Qsv (Fig. 2B through E). Despite observing an increase in p53 expression in F Media-cultured LXSN HFKs with HPV 16-Hygro Qsv infection (Fig. 2D), the increase in p16 protein and the presence of viral mRNA in this sample confirmed that HPV 16-Hygro Qsv infection was successful in this sample (Fig. 2D through E). While some differences are seen in NFX1-123 and viral gene expression between EpiLife and F Media culturing conditions, these reflect the natural variations in the expression of these genes. These results taken together validated the modulation of NFX1-123 expression and HPV 16-Hygro Qsv infection in HFKs.

**Fig 3:**
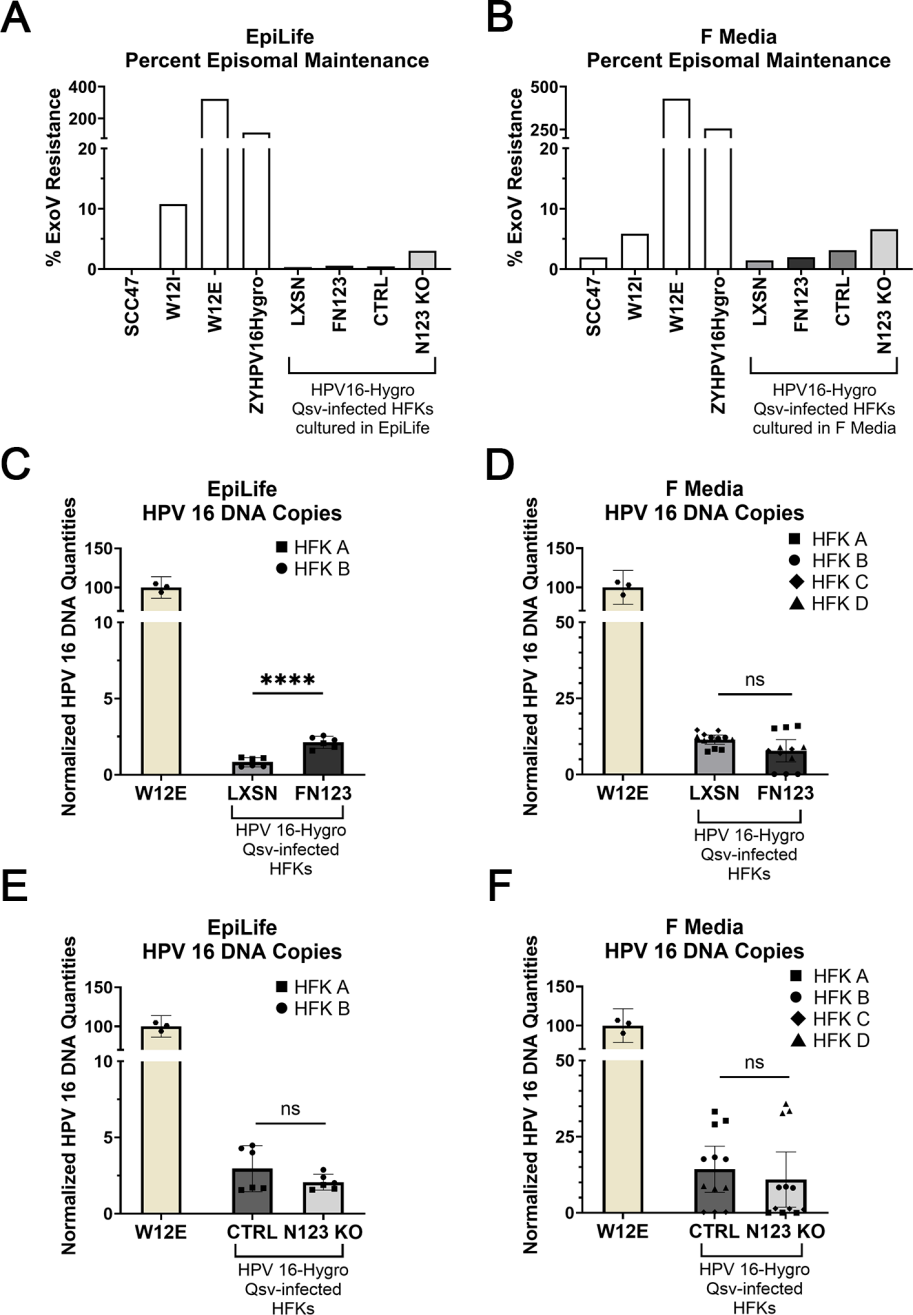
HPV 16-Hygro Qsv DNA integration and viral DNA abundance in HFKs. (**A and B**) Exonuclease V digestion of whole DNA extracts of Qsv-infected HFKs indicated that HPV 16-Hygro Qsv DNA integrated into the host genome after monolayer culturing and selection in (**A**) EpiLife and (**B**) F Media conditions. Percent Exonuclease V resistance was calculated using qPCR CT values of HPV 16 DNA (16E6) relative to endogenous diploid DNA amounts (18S). SCC47 and W12I served as control cell lines for integrated HPV 16 DNA, W12E served as a control cell line for episomally maintained HPV 16 DNA, and ZYHPV16Hygro is the plasmid used to generate the HPV 16-Hygro Qsv and functions as a control for circular viral DNA. (**C and D**) HPV 16-Hygro Qsv DNA copy numbers of FN123 and LXSN (**C**) HFK **A, B** cultured in EpiLife or (**D**) HFK** A–D** cultured in F Media relative to W12E cells. (**E and F**) HPV 16-Hygro Qsv DNA copy numbers of N123 KO and CTRL (**E**) HFK A, B cultured in EpiLife or (**F**) HFK **A–D** cultured in F Media relative to W12E cells. Individual points are technical replicates within each cell line, and error bars represent 95% confidence intervals across replicates from all cell lines. ****, *P* < 0.0001; ns, nonsignificant.

Next, we wanted to assess whether NFX1-123 expression impacted the maintenance and abundance of viral DNA after selection. We used four distinct biological backgrounds of HFKs (HFK A, B, C, and D) infected with HPV 16-Hygro Qsv and selected in either EpiLife or F Media with fibroblast feeders in monolayer culture conditions (Fig. 2A). While all four HFK backgrounds successfully came through selection in F Media, only two HFK lines (HFK A and B) did so in EpiLife. To assess viral genome status, we utilized an Exonuclease V (ExoV) assay, which digests linear DNA while leaving circular DNA intact (26). We found that all HFKs had HPV 16-Hygro Qsv DNA integrated into host chromosomal DNA, regardless of NFX1-123 status or culture methodology (Fig. 3A and B). When cultured in EpiLife, FN123, compared to LXSN HFKs, demonstrated an increase in viral DNA amounts amplified in HFKs A and B (Fig. 3C); while a decrease in viral DNA in N123 KO compared to CTRL HFKs was observed, these differences did not reach statistical significance (Fig. 3E).

When HFKs were cultured in F Media with fibroblast feeders, there were greater copies of HPV 16-Hygro Qsv DNA when compared to EpiLife cultures, normalized to W12E HPV 16 DNA abundance (Fig. 3C through F). When averaging all four HFK backgrounds, there was variability in viral DNA abundance that did not correlate with NFX1-123 expression (Fig. 3D and F). However, in three of the four HFK backgrounds, N123 KO had decreased viral DNA quantities when compared to its own CTRL (Fig. 3F). Taken together, it appeared that NFX1-123 modulation may have shifted the abundance of HPV 16-Hygro Qsv DNA in monolayer, non-differentiating culturing conditions.

### NFX1-123 knockout blunted HFK growth in organotypic raft cultures with and without HPV 16-Hygro Qsv infection

**Fig 4:**
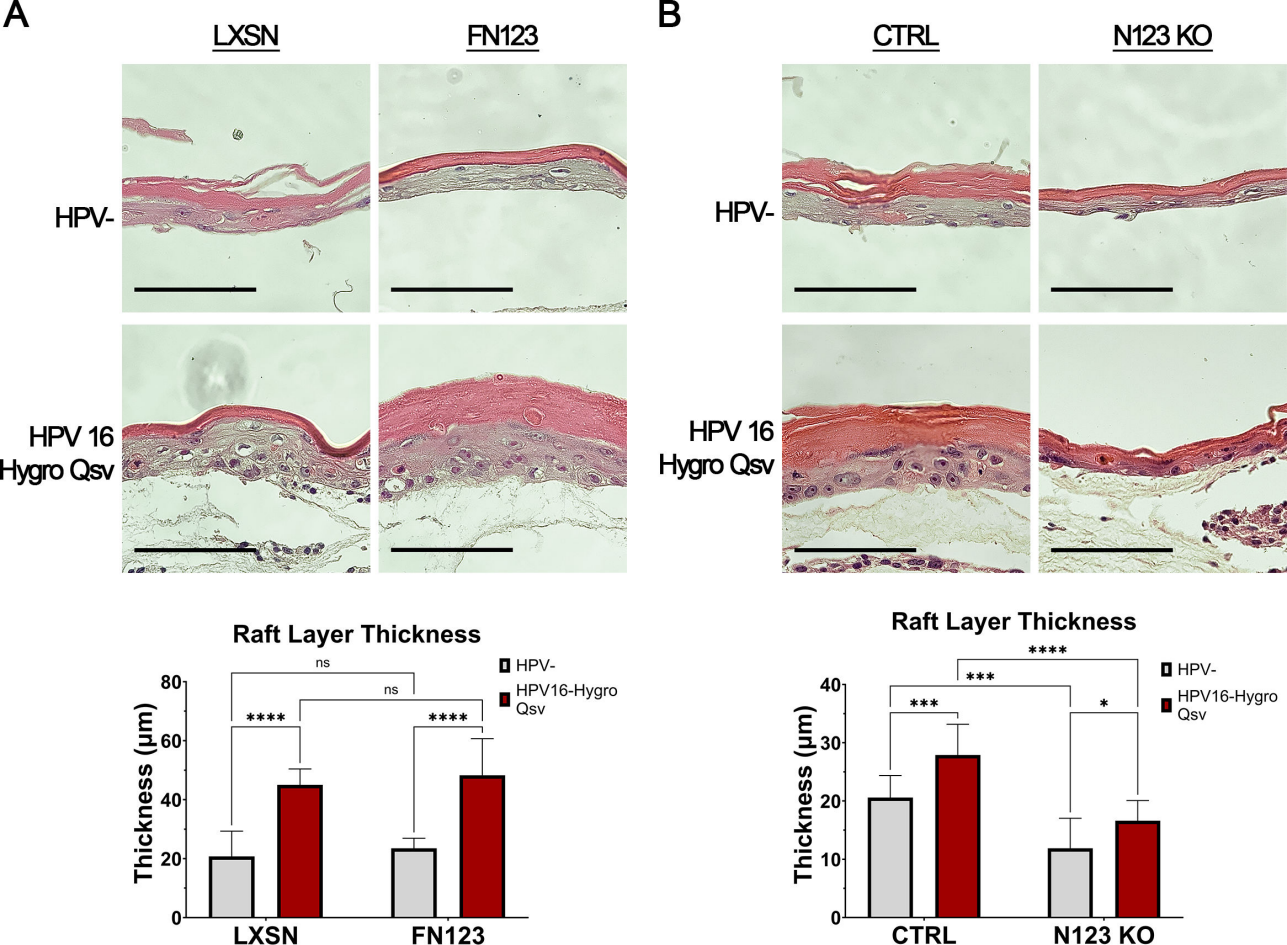
Stratified growth differences in organotypic rafts of HFKs with overexpressed, endogenous, and knocked out NFX1-123. (**A**) Organotypic raft cultures of FN123 and LXSN HFKs and (**B**) N123 KO and CTRL HFKs with and without HPV 16-Hygro Qsv infection were generated, formalin-fixed, paraffin-embedded, and stained with hematoxylin and eosin. Images were taken at 20× magnification on an ECHO REVOLVE microscope, and thickness was measured by calculating the distance of the non-cornified stratification basal to the start of the cornified layer, as reported on the REVOLVE microscope. Three fields of view of each raft were measured across five separate areas to generate 95% confidence intervals. One representative experiment from HFK A is shown from technical triplicate raft experiments conducted in two biologically independent HFK cell lines. Scale bar = 110 µm. *, *P* < 0.05, ***, *P* < 0.0005, ****, *P* < 0.0001, ns, nonsignificant.

We have demonstrated previously that NFX1-123 overexpression increases keratinocyte growth and differentiation when 16E6 is expressed (16, 18), but the effects of NFX1-123 expression on organotypic raft cultures in uninfected and early infection conditions have not been previously established. To mimic typical growth patterns of keratinocytes in stratified squamous epithelial tissue, we generated organotypic raft cultures of HFKs both with and without HPV 16-Hygro Qsv infection to elucidate if any growth or phenotypic differences would be seen in the stratified epithelium. FN123, LXSN, N123 KO, and CTRL HFKs were rafted as uninfected cells or after HPV 16-Hygro Qsv infection and selection. We first stained with hematoxylin and eosin to assess stratified growth and phenotypic differences, and we observed that all rafts of HFKs infected with HPV 16-Hygro Qsv consistently displayed enlarged, dysplastic-looking nuclei throughout the emulated epithelium compared to uninfected rafts (Fig. 4). While we did not observe quantifiable differences in non-cornified raft thickness between FN123 and LXSN HFKs, HPV 16-Hygro Qsv-infected rafts displayed thicker stratified epithelium compared to uninfected raft cultures (Fig. 4A). In N123 KO HFKs, there was decreased keratinocyte stratification and a lack of suprabasal keratinocytes in the intermediate phases of differentiation compared to CTRL HFKs, and these rafts did not achieve thickened stratified epithelium with HPV 16-Hygro Qsv infection to the same extent as CTRL rafts (Fig. 4B).

**Fig 5:**
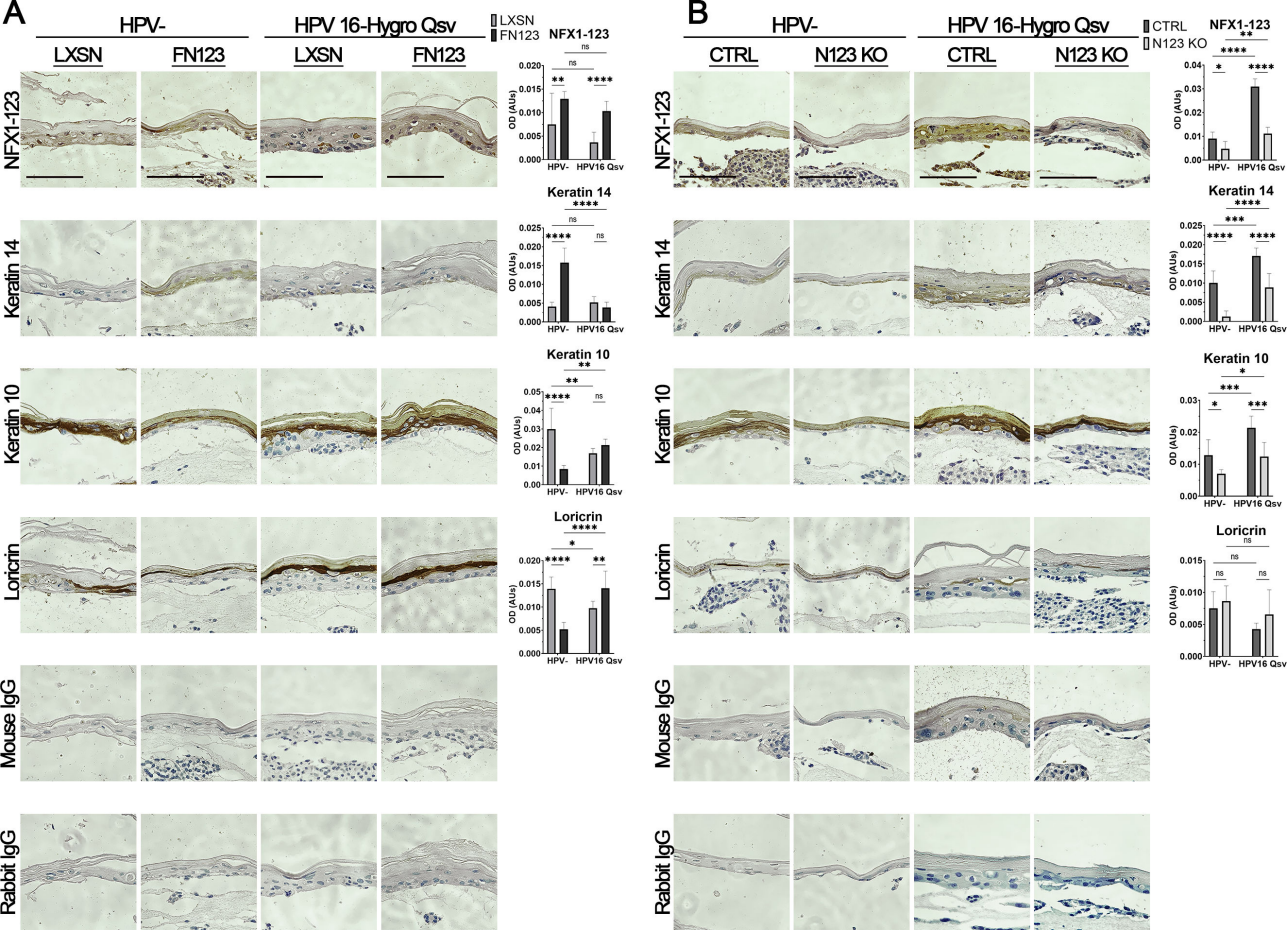
NFX1-123 regulates the expression of keratinocyte differentiation markers with HPV 16-Hygro Qsv in HFKs. (**A**) Organotypic raft cultures of FN123 and LXSN HFKs and (**B**) N123 KO and CTRL HFKs with and without HPV 16-Hygro Qsv infection were stained for protein expression of NFX1-123, Keratin 14, Keratin 10, and Loricrin via immunohistochemistry or stained using a rabbit or mouse polyclonal IgG as a control. Images were taken at 20× magnification on the REVOLVE microscope, and total staining intensity across five independent fields of view was calculated in ImageJ. Error bars represent 95% confidence intervals of DAB intensity across fields of view. One representative experiment from HFK A is shown from technical triplicate raft experiments conducted in two biologically independent HFK cell lines. Scale bar = 110 µm. *, *P* < 0.05, ***, *P* < 0.0005, ****, *P* < 0.0001, ns, nonsignificant.

We confirmed that modulation of NFX1-123 was maintained in raft cultures by immunohistochemical (IHC) staining (Fig. 5A through B, top row). To further elucidate how NFX1-123 expression affected keratinocyte growth and differentiation in raft cultures, we performed IHC staining for the basal cell marker Keratin 14, as well as differentiation markers Keratin 10 and Loricrin. While Ki67 staining was increased with HPV 16-Hygro Qsv infection of HFK rafts (data not shown), we chose to focus on basal and parabasal cell markers to define the stratification achieved in raft cultures with modulated NFX1-123 expression. Uninfected FN123 rafts with greater levels of NFX1-123 had increased staining for Keratin 14 and decreased staining for Keratin 10 and Loricrin compared to uninfected LXSN rafts; however, when HPV 16-Hygro Qsv was present, FN123 rafts had increased expression of Loricrin (Fig. 5A). This data confirmed that greater NFX1-123 gives rise to a more basal-like phenotype in keratinocytes without infection and required the presence of viral genes to drive increased expression of terminal differentiation markers when cells were actively differentiating. N123 KO HFK rafts had decreased staining for both the basal marker Keratin 14 and the differentiation marker Keratin 10 compared to CTRL rafts, both with and without HPV 16-Hygro Qsv (Fig. 5B). Taken with phenotypic growth and stratification thickness differences observed in Fig. 4B, these data indicated that knockout of NFX1-123 disrupted the typical differentiation and stratification process of keratinocytes in organotypic raft cultures, both without viral infection and in the context of an early HPV 16 infection.

### NFX1-123 correlated with HPV 16-Hygro Qsv DNA copies in differentiating conditions

**Fig 6:**
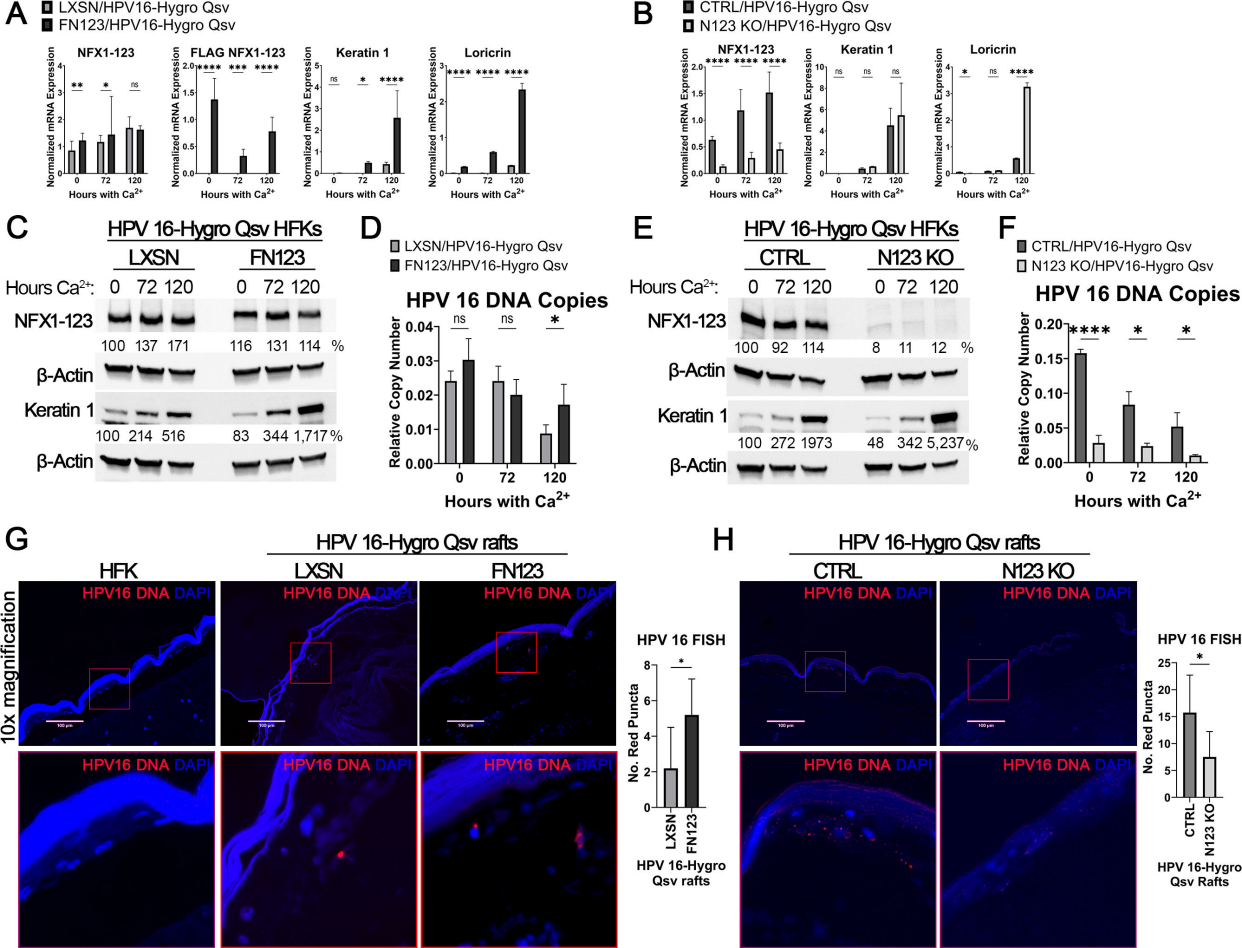
NFX1-123 expression correlates with HPV 16-Hygro Qsv DNA abundance in differentiated cells. (**A**) HPV 16-Hygro Qsv-infected FN123 and LXSN HFKs or (**B**) HPV 16-Hygro Qsv-infected N123 KO and CTRL HFKs were differentiated with high-dose calcium for 72 h or 120 h. NFX1-123, FLAG-tagged NFX1-123, Keratin 1, and Loricrin mRNA expression were measured in differentiated HFKs and undifferentiated controls and normalized to 36B4. (**C**) Immunoblotting of HPV 16-Hygro Qsv-infected FN123 and LXSN HFKs treated with high-dose calcium confirmed greater NFX1-123 in FN123 cell lines and increased Keratin 1 levels in differentiated cell lines. Densitometry of proteins (% expression) normalized to beta-actin and to undifferentiated LXSN expression is listed. (**D**) HPV 16-Hygro Qsv DNA copy numbers of differentiated and undifferentiated FN123 and LXSN HFKs. Error bars represent 95% confidence intervals across replicates from all cell lines. (**E**) Immunoblotting of HPV 16-Hygro Qsv-infected N123 KO and CTRL HFKs treated with high-dose calcium confirmed reduction of NFX1-123 in N123 KO cell lines and increased Keratin 1 levels in differentiated cell lines. Densitometry of proteins (% expression) normalized to beta-actin and to undifferentiated CTRL expression is listed. (**F**) HPV 16-Hygro Qsv DNA copy numbers of differentiated and undifferentiated N123 KO and CTRL HFKs. Error bars represent 95% confidence intervals across replicates from all cell lines. (**G**) *In situ* hybridization for HPV 16 DNA in FN123 and LXSN and (**H**) N123 KO and CTRL HPV 16-Hygro Qsv-infected raft cultures was performed using the Zyto*Fast* HPV type 16/18 probe and detected using Alexa Fluor-conjugated tyramide signal amplification. An uninfected HFK raft was also hybridized as a control. Images in the top row were taken at 10× magnification using DAPI/TXRED fluorescent overlay settings, and images in the bottom row are from the inset. The number of red fluorescent puncta was calculated across 10 fields of view in ImageJ. Error bars represent 95% confidence intervals from independent fields of view. One representative experiment from HFK A is shown *, *P* < 0.05, ***, *P* < 0.0005, ****, *P* < 0.0001, ns, nonsignificant.

Given the evidence that NFX1-123 plays a crucial role in keratinocyte differentiation (16) (Fig. 4 and 5), we hypothesized that modulation of NFX1-123 would impact the maintenance and amplification of HPV DNA in differentiating conditions. We induced differentiation in HPV 16-Hygro Qsv-infected HFKs using high-dose calcium in monolayer culture, and we serially assessed viral copy numbers using qPCR. Although increases in total NFX1-123 mRNA and protein were modest in FN123 HFKs compared to LXSN HFKs with differentiation (Fig. 6A and C), we confirmed the presence of exogenous FLAG-tagged NFX1-123 in differentiated cells by RT-qPCR (Fig. 6A). Similarly, we confirmed reduction of total NFX1-123 mRNA and protein in N123 KO cells compared to CTRL HFKs by RT-qPCR and western blot (Fig. 6B and E). In all treated cell lines, protein levels of the differentiation marker Keratin 1 rose at 72 h and 120 h when compared to 0 h (Fig. 6C and E). Induction of differentiation was further confirmed by increased mRNA of Keratin 1 and Loricrin, with greatest increases seen at 120 h of treatment (Fig. 6A and B). HPV 16-Hygro Qsv DNA copy numbers were assessed with differentiation. Regardless of viral integration status, at 120 h of calcium exposure, FN123 HFKs had increased relative HPV 16-Hygro Qsv abundance compared to LXSN HFKs (Fig. 6D). In the N123 KO HFKs, HPV 16-Hygro Qsv DNA abundance is decreased compared to CTRL cells, both in undifferentiated and differentiated conditions (Fig. 6F). This data confirmed that FN123 HFKs with high expression of NFX1-123 supported increased HPV 16-Hygro Qsv DNA abundance compared with LXSN HFKs, and that knockout of NFX1-123 blunted viral DNA amounts with differentiation compared to CTRL cells.

We next investigated viral DNA abundance and localization using fluorescent *in situ* hybridization (FISH) in HPV 16-Hygro Qsv-infected HFKs in raft cultures (Fig. 6G and H). FN123 rafts had increased detectable numbers of puncta, indicating an elevated number of HPV 16-Hygro Qsv genomes compared to LXSN rafts (Fig. 6G). N123 KO rafts had decreased overall fluorescence and fewer distinct puncta compared to CTRL rafts (Fig. 6H). These results indicated that NFX1-123 expression was required for typical levels of HPV 16-Hygro Qsv genomes in differentiated keratinocytes, as reflected in monolayer differentiation conditions and raft cultures, and greater expression of NFX1-123 led to higher amounts of HPV 16 genomes.

### NFX1-123 knockout reduced episomal HPV 16 copy numbers in W12E cells

**Fig 7:**
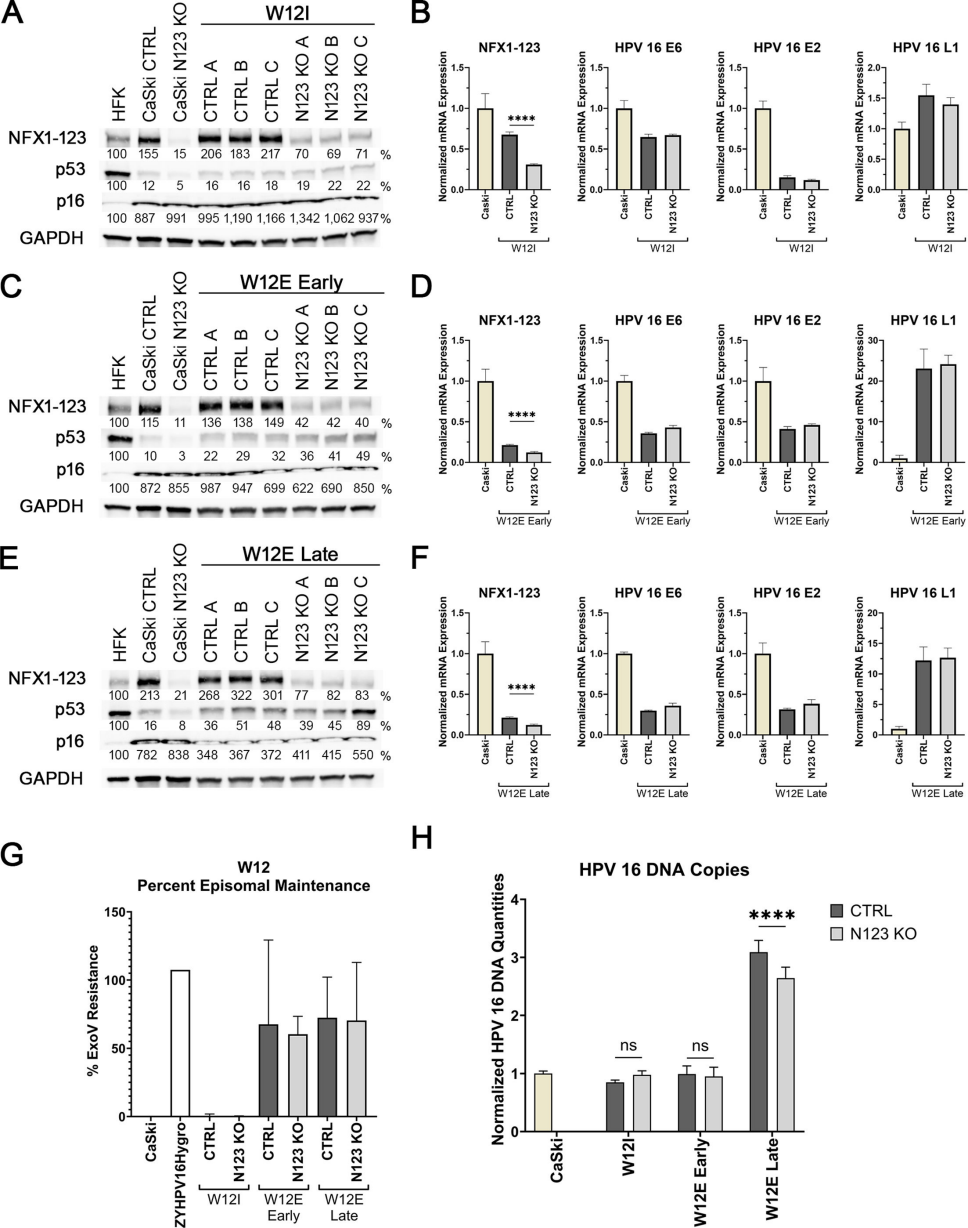
Validation of NFX1-123 knockout, HPV 16 gene expression, and viral DNA maintenance in W12 cell lines. (**A**) Immunoblotting of W12I, (**C**) W12E early, and (**E**) W12E late CTRL and N123 KO cells confirmed decreased NFX1-123 protein in N123 KO cell lines compared to CTRL. Immunoblotting for p53 and p16 confirmed 16E6 and 16E7 protein activity, respectively. NFX1-123 knockdown was conducted in technical triplicate (termed A, B, and C). CaSki CTRL and N123 KO cell lines are included as a control for knockout of NFX1-123, reduced p53, and increased p16 levels relative to an uninfected HFK cell line. Densitometry of proteins (% expression) normalized to GAPDH and to uninfected HFK expression is listed. (**B, D, and F**) NFX1-123, HPV 16E6, HPV 16E2, and HPV 16L1 mRNA expression were measured by RT-qPCR and normalized to 36B4 and to CaSki gene expression. Error bars represent 95% confidence intervals of three technical triplicates. (**G**) Exonuclease V digestion of whole DNA extracts of W12I and W12E early and late CTRL and N123 KO cell lines indicated that viral DNA was integrated in W12I cell lines and remained episomal in early and late W12E cells, regardless of NFX1-123 status. Percent Exonuclease V resistance was calculated using qPCR CT values of HPV 16 DNA (16E6) relative to endogenous diploid DNA amounts (18S). CaSki served as a control cell line for integrated HPV 16 DNA, while ZYHPV16Hygro is the plasmid used to generate HPV 16-Hygro Qsv and functions as a control for circular viral DNA. (**H**) HPV 16 DNA copy numbers of N123 KO and CTRL W12I and W12E early and W12E late cells relative to CaSki cells. Error bars represent 95% confidence intervals of technical triplicates across three CTRL and N123 KO replicates. ****, *P* < 0.0001; ns, nonsignificant.

Our studies in HFKs modeled a cellular background for early HPV infection and establishment. To determine whether NFX1-123 modulation influenced HPV 16 DNA maintenance in models of persistent infection, we utilized W12 cells, a cervical cell line containing either episomal (W12E) or integrated (W12I) copies of HPV 16 DNA. In technical triplicates (A, B, and C), W12E and W12I cells were transduced to knockout NFX1-123 (N123 KO) or retain NFX1-123 expression with a control vector (CTRL) (Fig. 7). NFX1-123 protein was consistently decreased in W12I N123 KO (Fig. 7A) and in W12E N123 KO cells (Fig. 7C and E). With HFKs as an HPV 16-negative control and CaSki (N123 KO or CTRL) as an HPV 16-positive control, p53 was decreased, and p16 was increased in CTRL and N123 KO W12I and W12E cells (Fig. 7A, C, and E). To determine whether reduced NFX1-123 could be sustained in the N123 KO cells, W12E N123 KO, and CTRL cells were maintained in culture (early versus late), and NFX1-123 protein did not rebound in W12E late cells (Fig. 7E). N123 KO decreased NFX1-123 mRNA in W12I and in both W12E early and late cells compared to CTRL cells (Fig. 7B, D, and F). HPV genes 16E6, 16E2, and 16L1 were all confirmed by RT-qPCR, and we found higher 16E2 and 16L1 mRNA amounts in W12E cells (Fig. 7D and F) compared to W12I cells (Fig. 7B), which mirrored their episomal (W12E) and integrated (W12I) viral DNA status. Virtually, no episomal HPV 16 DNA was detected in CTRL and N123 KO W12I cells (Fig. 7G). In early and late W12E cells, 75% of the HPV 16 DNA was episomal, and N123 KO did not impact the fraction of HPV DNA that was episomally maintained (Fig. 7G). Next, we quantified total copy numbers (integrated or episomal) of HPV 16 DNA in CTRL and N123 KO W12 cells. In our W12I and early W12E cells, we observed a low relative copy number of HPV 16 DNA with no difference in viral copy number when comparing CTRL to N123 KO cells (Fig. 7H). However, in late W12E cells, HPV 16 DNA was increased relative to early W12E cells, and N123 KO blunted this increase compared to CTRL W12E cells (Fig. 7H). These data indicated that a reduction of NFX1-123 decreased the episomal copies of HPV 16 DNA in monolayer cultures over time.

### NFX1-123 knockout decreased dysplastic phenotypes in W12E cells in organotypic raft cultures

**Fig 8:**
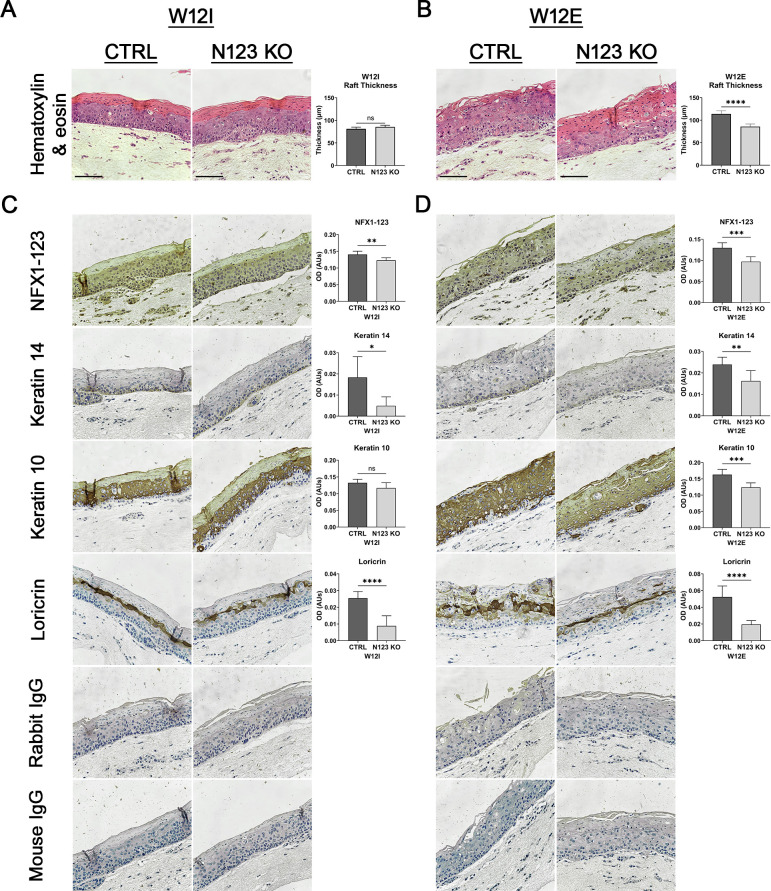
Knockout of NFX1-123 in W12 rafts resulted in decreased growth and dysplastic phenotypes compared to control rafts. (**A and B**) Organotypic raft cultures of N123 KO and CTRL (**A**) W12I and (**B**) W12E late cells were generated, formalin-fixed and paraffin-embedded, and stained with hematoxylin and eosin. Images were taken at 20× magnification on the REVOLVE microscope, and thickness was measured by calculating the distance of the non-cornified stratification to the start of the cornified layer, as reported on the REVOLVE scope. Three fields of view were measured across five separate areas to generate 95% confidence intervals. Scale bar = 110 µm. (**C**) W12I and (**D**) W12E N123 KO and CTRL organotypic raft cultures were stained for protein expression of NFX1-123, Keratin 14, Keratin 10, and Loricrin via immunohistochemistry or stained using a mouse or rabbit IgG as a control. Images were taken at 20× on the REVOLVE microscope, and total staining intensity across five fields of view was calculated in ImageJ. Error bars represent 95% confidence intervals of DAB intensity across fields of view. *, *P* < 0.05, **, *P* < 0.005, ***, *P* < 0.0005, ****, *P* < 0.0001, ns, nonsignificant.

To compare whether knockout of NFX1-123 would affect W12 cellular differentiation and stratification in a similar manner to HPV 16-Hygro Qsv infected HFKs (Fig. 4), W12I and W12E late CTRL and N123 KO cells were grown in raft cultures. Consistent with our immunoblot data, total NFX1-123 staining intensity was reduced in N123 KO W12I and W12E rafts compared to CTRL rafts (Fig. 8C and D, top row). Interestingly, in W12E N123 KO rafts, knockout of NFX1-123 led to decreased full thickness, non-cornified stratification compared to CTRL rafts (Fig. 8B). However, this decrease in raft thickness was not observed in W12I N123 KO rafts compared to W12I CTRL rafts (Fig. 8A). Despite there being no changes to the thickness of W12I rafts with N123 KO, Keratin 14 staining and Loricrin staining were significantly decreased in W12I N123 KO rafts compared to CTRL rafts (Fig. 8C), indicating that the reduction of NFX1-123 is disrupting both the basal and differentiation phenotypes of W12I cells within these rafts. In the W12E N123 KO rafts, we also observed a decrease in nucleated suprabasal cells and fewer dysplastic changes compared to W12E CTRL rafts, but this decrease in dysplastic-looking nuclei with NFX1-123 knockout was not seen to the same extent in W12I N123 KO rafts (Fig. 8A and B). Similar to HPV 16-Hygro Qsv-infected HFK rafts, we observed phenotypic differences in the non-cornified layer of raft cultures when comparing W12E CTRL and N123 KO rafts, and these changes mirrored differences in the staining intensities of Keratin 14, Keratin 10, and Loricrin (Fig. 8D). W12E N123 KO rafts had less intense staining and a more limited area of positive staining for the differentiation markers Keratin 10 and Loricrin compared to W12E CTRL rafts, and had significant decreases in Keratin 14 staining (Fig. 8D), indicating that NFX1-123 knockout was disrupting typical keratinocyte phenotypes of W12E cells at both early and late stages of differentiation and in the basal compartment of W12E rafts. These results demonstrated that NFX1-123 knockout disturbed the expression of typical keratinocyte markers at various stages of differentiation of W12E and W12I cells in organotypic raft cultures, although significant thickness differences were only seen in W12E rafts.

### NFX1-123 knockout blunted HPV 16 copy numbers in differentiated cultures of W12E cells

**Fig 9:**
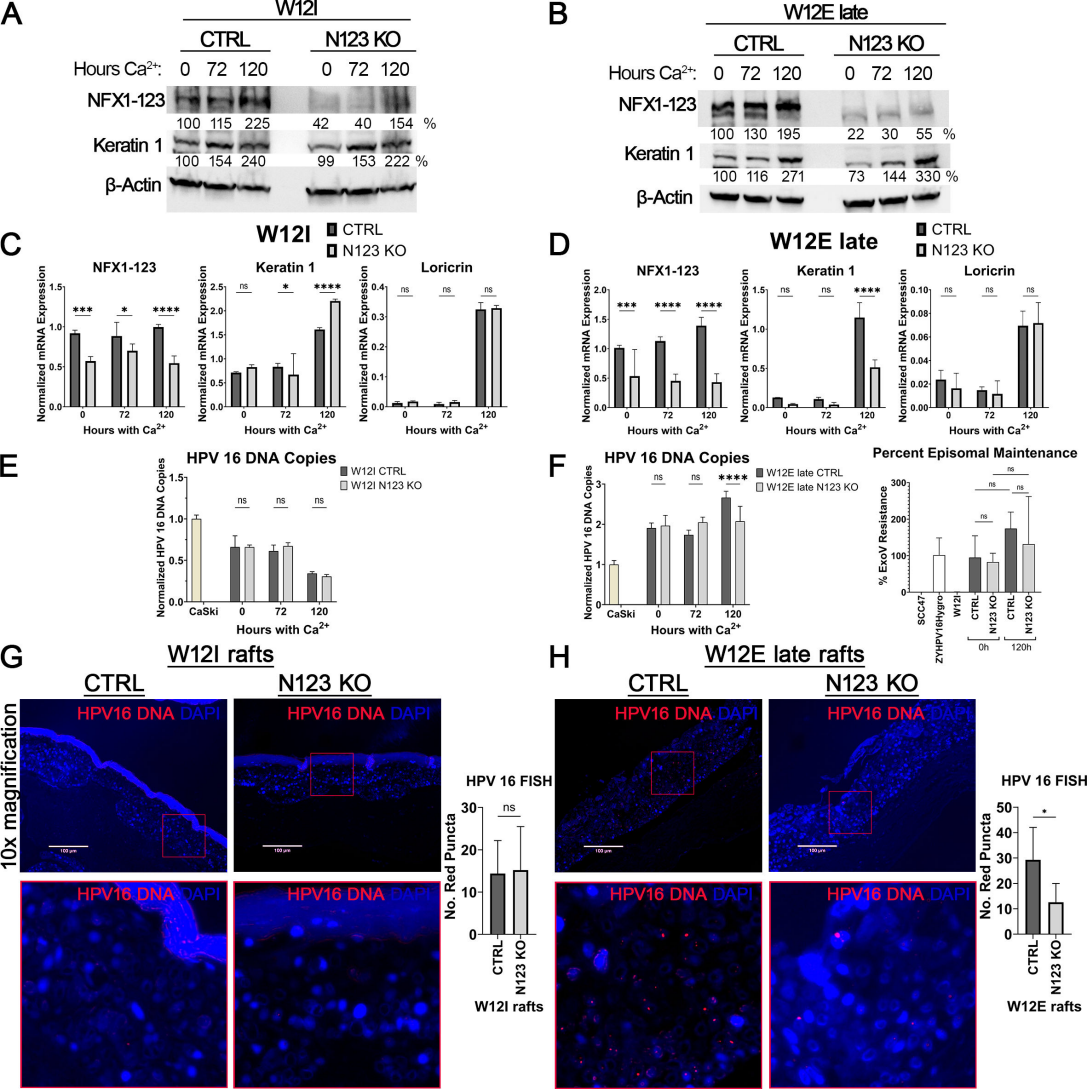
NFX1-123 expression correlates with HPV 16 DNA copy numbers in differentiated W12E cells. (**A**) W12I N123 KO and CTRL or (**B**) W12E late N123 KO and CTRL cells were differentiated with high-dose calcium for 72 h or 120 h. Immunoblotting of cells treated with high-dose calcium confirmed the reduction of NFX1-123 in N123 KO cell lines, as well as increased Keratin 1 levels in differentiated cell lines. Densitometry of proteins (% expression) normalized to beta-actin and to undifferentiated CTRL expression is listed. (**C and D**) NFX1-123, Keratin 1, and Loricrin mRNA expression were measured in differentiated and undifferentiated (**C**) W12I N123 KO and CTRL cells and (**D**) W12E late N123 KO and CTRL cells and normalized to 36B4. (**E and F**) HPV 16 DNA copy numbers of differentiated and undifferentiated (**E**) W12I N123 KO and CTRL cells and (**F**) W12E late N123 KO and CTRL cells. (F, right) Percent episomal maintenance of W12E CTRL and N123 KO cells with 120 h of differentiation is shown. Error bars represent 95% confidence intervals across replicates from all cell lines. (**G and H**) *In situ* hybridization for HPV 16 DNA in (**G**) W12I and (**H**) W12E late N123 KO and CTRL raft cultures was performed using the Zyto*Fast* HPV type 16/18 probe and detected using Alexa Fluor-conjugated tyramide signal amplification. Images in the top row were taken at 10× magnification using DAPI/TXRED fluorescent overlay settings, and images in the bottom row are from the inset. The number of red fluorescent puncta was calculated across 10 fields of view in ImageJ. Error bars represent 95% confidence intervals from independent fields of view. *, *P* < 0.05, **, *P* < 0.005, ***, *P* < 0.0005, ****, *P* < 0.0001, ns, nonsignificant.

Since NFX1-123 expression appeared to be required for typical differentiation in W12E raft cultures, we wanted to explore whether NFX1-123 knockout would impact HPV 16 DNA copy numbers. We induced differentiation with high-dose calcium in monolayer cultures of W12I and W12E late cells. We confirmed reduction of total NFX1-123 mRNA and protein in N123 KO cells compared to CTRL W12I and W12E cells by RT-qPCR and western blot across all time points (Fig. 9A and D). Compared to 0 h, Keratin 1 protein increased at 72 h and rose further at 120 h (Fig. 9A and B). Induction of differentiation was further confirmed by increased Keratin 1 and Loricrin mRNA at 120 h (Fig. 9C and D). Total DNA was isolated at each time point, and HPV 16 DNA copy numbers were assessed with differentiation. As expected, we did not observe any significant differences in the HPV 16 DNA copy numbers in W12I N123 KO cells compared to W12I CTRL cells with or without calcium treatment (Fig. 9E). Interestingly, we observed a decrease in total detectable HPV 16 DNA levels in samples with calcium treatment in W12I cells, and this trend was mirrored in our HPV 16-Hygro Qsv-infected HFKs treated with calcium (Fig. 6C and F). In W12E cells, HPV 16 DNA copy numbers increased at 120 h of calcium treatment, indicating that viral genome amplification occurred with differentiation, and this amplification is blunted in N123 KO cells (Fig. 9F, left graph). ExoV digestion confirmed that HPV 16 DNA was maintained episomally at 0 h and 120 h in W12E CTRL and N123 KO cells (Fig. 9F, right graph). To determine whether the HPV DNA differences seen in monolayer culture also occurred in raft cultures, we conducted HPV 16 FISH staining of W12I and W12E late cells. Consistent with low viral copy numbers observed in W12I monolayer cultures, W12I rafts had modest HPV 16 DNA staining; there was also no significant difference in viral genome detection in N123 KO W12I rafts when compared to CTRL (Fig. 9G). W12E CTRL rafts, however, contained a greater number of distinct puncta relative to W12I rafts, and N123 KO W12E rafts had less fluorescence with fewer distinct puncta throughout basal and differentiated layers compared to W12E CTRL rafts (Fig. 9H). Taken together, these data indicated that a reduction of NFX1-123 decreased the episomal copies of HPV 16 DNA in both monolayer and organotypic raft cultures.

## DISCUSSION

HPV 16 initiates infection in basal keratinocytes of stratified squamous epithelium and utilizes host cell machinery and the tissue architecture to regulate the viral life cycle, amplify viral genomes, and sustain a productive infection (6). We have previously shown that the HPV 16 E6 oncoprotein binds directly to the endogenous host protein NFX1-123 to manipulate several host cell processes beneficial to the viral life cycle, including promotion of simultaneous proliferation and differentiation (11, 15, 16, 19, 20). Here, we describe for the first time the requirement for full expression of NFX1-123 to establish and maintain HPV viral DNA at high copy numbers to support persistent infections and dysplasias.

To investigate whether NFX1-123 expression impacted the initial stages of an HPV infection, we utilized a pseudovirus system paired with an established ECM infection methodology (22). The HPV 16 capsid can package HPV DNA, but it can also package non-viral expression plasmids if sized appropriately. This flexibility provides an effective method to study the first few hours after initial HPV infection, prior to detectable viral gene expression and selection. Using an mCherry plasmid encapsidated in the HPV 16 capsid shell, an mCherry pseudovirus (Psv), we found no differences in mCherry Psv infectivity in HFKs with overexpressed, endogenous, or knocked out NFX1-123, and, regardless of NFX1-123 status, the percentage of infected HFKs increased between 24 and 48 h after infection with mCherry Psv. This supports our prior findings that NFX1-123 is primarily a cytoplasmic protein (11) and likely would not function at the cell surface to impact viral entry into HFKs or early episomal gene expression.

To model the initial steps of an HPV 16 infection after entry, we employed an HPV 16-Hygro Qsv to infect HFKs. Qsv aids in studying the initial steps of HPV infection by being able to quickly produce mature virions at large viral titers and mimicking natural pathways of infection. An additional advantage of our HPV 16-Hygro Qsv system is the hygromycin resistance gene, which allows for the selection of infected cells. It is important because HPVs establish their infections with low viral genome copies and gene expression (6, 7), making it difficult to quantify the efficiency of infection of native HPV in monolayer culture conditions. Others have found that the addition of antibiotic resistance genes to human papillomavirus genomes can be used to study the early stages of infection without affecting early viral replication (27); therefore, selection after infection aids in creating a more homogeneous cell culture.

To assess whether baseline NFX1-123 abundance altered HPV 16-Hygro Qsv genome copy numbers, weeks after infection and hygromycin selection, we assessed the state and abundance of Qsv DNA (26). Despite varying the manner by which HFKs were grown in culture, HPV 16-Hygro Qsv DNA integrated into host genomes in all HFK backgrounds tested. One potential reason for this was that infected HFKs required an average of 3 weeks in selection media before quantifying viral DNA copy numbers. As long-term *in vitro* passaging of cells harboring HPV DNA increases the likelihood of viral genome integration events (28, 29), we hypothesize that this extended time in continuous hygromycin selection allowed for HPV 16-Hygro Qsv genomes to integrate into host DNA. Additionally, extended treatment of HFKs with hygromycin may have acted as a selective pressure, leading to an outgrowth of cell population subsets, where HPV 16-Hygro Qsv integration had already occurred, masking any heterogeneity in the infected HFK populations, where episomal Qsv genomes initially may have been present and potentially limiting the model’s applicability to early infection.

Despite observing consistent integration of HPV 16-Hygro Qsv genomes into host DNA after extended time in culture, we expected that our HPV 16-Hygro Qsv model was permissive of the initial establishment phase of HPV DNA replication prior to integration (7). While we saw a range in HPV DNA copy number across our biologically independent HFK backgrounds, regardless of the amount of NFX1-123 expressed, reflecting the variability in biologic backgrounds, we did observe that monolayer HFKs co-cultured in F Media with fibroblast feeders tended to harbor greater HPV 16-Hygro Qsv DNA amounts when compared to HFKs cultured in EpiLife, likely reflecting that HFKs in F Media were initially able to support greater amounts of episomal HPV DNA that then became integrated.

While we observed inconsistent HPV 16-Hygro Qsv DNA copy numbers in undifferentiated monolayer cultures of HFKs, we consistently observed that full expression of NFX1-123 was required to form typical organotypic raft cultures and maintain viral copy numbers in differentiating conditions, and overexpression of NFX1-123 may augment these viral copy numbers. Our laboratory has previously observed that there is a range of endogenous NFX1-123 expression in stratified squamous epithelium, both across patient samples and within the basal layer (15, 16). Here, we have identified for the first time that NFX1-123 expression is required for keratinocyte stratification and differentiation in raft cultures, both with and without the context of an HPV 16 infection. The result that NFX1-123 is required for stratification of uninfected tissues indicates that this protein plays an essential role in typical keratinocyte differentiation.

Prior studies from our laboratory and others have found that high expression of NFX1-123 offers a growth advantage to 16E6-expressing HFKs in monolayer cultures (11, 15, 18, 19) and that increased levels of NFX1-123 were associated with dysplastic stratified epithelial tissue architecture in both raft cultures and patient tissue samples (15, 18). In addition to phenotypic observations of dysplastic growth, HPV DNA load is also considered an ancillary marker for a persistent HPV infection and the likelihood for progression to cancer (30, 31). In our HPV 16-Hygro Qsv HFK raft cultures, we observed a decrease in stratification thickness in N123 KO rafts compared to CTRL rafts, and this correlated with a decrease in viral DNA detection by FISH, indicating that NFX1-123 may contribute to HPV 16 infection persistence and oncogenic potential. Furthermore, FN123 rafts of HFKs infected with HPV 16-Hygro Qsv displayed increased viral copy numbers with differentiation compared to LXSN rafts. Taken as a collective, these results indicate that keratinocytes with high expression of NFX1-123 may harbor HPV 16 infections better than those that are initially established in basal cells with lower levels of NFX1-123.

Extending the role of NFX1-123 in growth, differentiation, and HPV DNA amounts from HFKs to dysplastic cervical cells that have maintained HPV 16 DNA, we again found that NFX1-123 was required to form full-thickness stratified squamous epithelium in raft cultures of W12E cells. Not only were there decreased stratification and fewer dysplastic cells in N123 KO W12E rafts compared to CTRL W12E rafts, but HPV 16 DNA copy numbers also fell, and viral DNA detection was reduced in W12E late cells. These results are consistent with our prior findings that NFX1-123 knockdown in W12E cells blunted HPV 16 L1 protein expression (16) and thus is required for the full completion of the late stages of the viral life cycle.

These studies utilizing models of early and persistent HPV 16 infections indicated that NFX1-123, by supporting keratinocyte growth and differentiation, also influences the maintenance phase of the HPV 16 life cycle. We showed that NFX1-123 was crucial for the typical stratification and differentiation of keratinocytes, and that elevated levels of NFX1-123 increase keratinocyte growth in the basal layer. During both early and persistent HPV 16 infections, NFX1-123 was required for keratinocyte differentiation, and these disruptions in stratified epithelial tissue architecture were associated with decreased episomal HPV 16 copy numbers. As endogenous NFX1-123 expression varies across and within normal cervical samples, these data have implications that people who have higher levels of NFX1-123 may better harbor and maintain an HPV infection, increasing their risk for neoplastic development, and those with lower NFX1-123 expression may not. Additionally, our studies in W12 cells indicated that sustained reduction of NFX1-123 in cells already harboring persistent HPV 16 infections can reduce their growth and episomal viral copy number. NFX1-123 expression is critical for differentiation and supports keratinocyte differentiation, stratification, and HPV 16 DNA episomal maintenance, which are key requirements for persistent productive HPV infections. These findings encourage further investigation of NFX1-123 as a potential therapeutic target for HPV 16-positive cervical infection and disease.

## MATERIALS AND METHODS

### Plasmids

p16sheLL expressing codon optimized HPV 16 L1 and L2 capsid proteins for pseudovirus production and the mCherry plasmid were a kind gift from Dr. Timra Gilson and Dr. Elliot Androphy. pMC16-Hygro was cloned by replacing the neomycin resistance cassette in PMC16-Neo (also a kind gift from Dr. Timra Gilson and Dr. Elliot Androphy) with a hygromycin resistance cassette from pBABE-hygro (Addgene, 1765-DNA). Primers were designed to encompass the SV40 promoter and hygromycin resistance gene from pBABE-hygro while adding a Bsu36I restriction site to the 5′ end of the promoter (F- AGCAAGCCTAAGGGTGTGTCAGTTAGGGTGTGGAAAGT and R- CTATTCCTTTGCCCTCGGACGA). The neomycin resistance gene was released from pMC16-Neo at SmaI and Bsu36I restriction sites, and the PCR-amplified hygromycin cassette was ligated into the plasmid backbone at the same sites to create pMC16-Hygro. Sequencing confirmed successful replacement of the neomycin cassette with the hygromycin cassette and that no mutations to the HPV 16 genome were incorporated during cloning. To produce HPV minicircles for Qsv production, pMC16-Hygro was recombined in ZYCY10P32S2T *E. coli* (System Biosciences, Palo Alto, CA, US, #MN900A-1), and the resulting recombined, package-ready plasmid is referred to as ZYHPV16Hygro. The FLAG-tagged NFX1-123 (FN123), LXSN vector control (LXSN), CRISPR-Cas9-mediated NFX1-123 knockout (N123 KO), and knockout control vector (targeting a non-mammalian gene target; CTRL) have been described previously (11, 32).

### Cell culture

Primary human foreskin keratinocytes (HFKs) were derived from anonymous neonatal foreskins and cultured in EpiLife medium supplemented with 60 µM calcium chloride (Gibco, ThermoFisher Scientific, Waltham, MA, US, MEPI500CA) and human keratinocyte growth supplement (HKGS; Gibco, ThermoFisher Scientific, Waltham, MA, US, S0015) or F-Media (33). HPV 16-positive cervical epithelial cells with either integrated (W12I) or episomal (W12E) viral genomes were grown in F-Media. CaSki and SCC47 cells, HPV 16-positive, integrated cervical and head and neck cancer cell lines, respectively, were cultured in complete DMEM medium (Gibco, ThermoFisher Scientific, Waltham, MA, 11965092) with sodium pyruvate, 10% fetal bovine serum (FBS), and 1% penicillin and streptomycin (P/S). Cells grown in F-Media were co-cultured with 1 million J2 3T3 fibroblast feeder cells pretreated with 6 µg/mL Mitomycin C (Sigma-Aldrich, St. Louis, MO, US, M7949) for 3–4 h. Stable J2 3T3s were generated by transfection with resistance genes for hygromycin (pBABE-hygro), puromycin (pBABE-puro), or neomycin/G418 (a kind gift from Dr. Timra Gilson and Dr. Elliot Androphy). When harvesting cells cultured in F-Media with feeders, the cells were briefly trypsinized and washed with DPBS (Gibco, ThermoFisher Scientific, Waltham, MA, 14190144) to remove feeders. The cells were then trypsinized again to harvest the cells of interest. W12E early cells were harvested for analysis six passages post-infection with lentivirus. W12E late cells were harvested 10 passages post-infection.

### Retrovirus/lentivirus production and transduction

293T cells were cultured in complete DMEM medium with sodium pyruvate, 10% FBS, and 1% P/S. 293T cells were grown to confluency on 15 cm dishes. The FLAG-tagged NFX1-123 retrovirus (FN123) and its control (LXSN) were produced in 293T cells by a transient vesicular stomatitis virus G-pseudotyped virus (VSV-G) protocol as previously described (34). The NFX1-123 knockout lentivirus (N123 KO) and its control (CTRL) were also produced as previously described (11). Briefly, the constructs were transfected into 293T cells using FuGENE6 (Promega, Madison, WI, US, E2692). After 24 h of transfection, media containing produced virus was serially collected four times (twice daily for FN123 and LXSN virus and once daily for N123 KO and CTRL virus), concentrated by ultracentrifugation using a SW28 rotor for 90 min at 16,000 rpm and 4°C, mixed with Polybrene (8 µg/mL) (Sigma-Aldrich, St. Louis, MO, US, TR-1003), and incubated with 50%–60% confluent HFK, W12, or CaSki cells. After 3 h of incubation at 37°C, the medium was replaced with EpiLife, F-Media, or DMEM. The cells were expanded 24 h post-transduction and placed under neomycin/G418 or puromycin selection 48 h post-transduction.

### HPV16-Hygro quasivirus and mCherry pseudovirus production

293TTF cells were cultured in complete DMEM medium with sodium pyruvate, 10% FBS, and 1% P/S. 293TTF cells were seeded at 4.5 × 10^6^ cells per plate onto 10 cm dishes. After 24 h of seeding, the cells were transfected with the plasmid encoding the HPV 16 capsid (p16sheLL) and either the mCherry plasmid or ZYHPV16Hygro using Lipofectamine 3000 reagent (Invitrogen, ThermoFisher Scientific, Waltham, MA, US, L3000015). The day after transfection, the media was changed, and 1% 100× MEM non-essential amino acids (NEAA; Gibco, ThermoFisher Scientific, Waltham, MA, US, 11140050) was added to the cells. After 48 h of transfection, cells were pelleted and gently resuspended in 1.5 cell pellet volume equivalents of viral maturation buffer (0.5% Triton X-100, 10 mM MgCl_2_, 5 mM CaCl_2_, and PBS), then incubated at 37°C for 2 days in siliconized microcentrifuge tubes. Lysates were spun at 7,000 rpm for 5 min and soluble viral stock was stored at 4°C for up to 1 month.

### Quasivirus and pseudovirus quantification

Once Qsv/Psv was collected, it was quantified by first digesting non-encapsidated DNA. An amount of 5 µL of virus was mixed in a 50 µL reaction with 1 µL of benzonase (Sigma-Aldrich, E8263), 2 µL 50 mM MgCl_2_, and 15 mM Tris-HCl, pH 7.5, and incubated for 30–45 min at 37°C. This mixture was subsequently heated to 55°C, and 1 µL of 0.5 M EDTA, pH 8.0, was added to slow the benzonase. An amount of 2 µL of 10 mg/mL proteinase K (Sigma-Aldrich, St. Louis, MO, US, 3115879001) was added to digest the capsid proteins and incubated for 30–45 min at 55°C. 3 µL of 0.1 M phenylmethanesulfonyl fluoride solution in ethanol (Sigma-Aldrich, St. Louis, MO, US, 93482) was added. For quantification by qPCR, digested virus was diluted 1:15 in molecular biology grade nuclease-free water and run alongside a standard curve with primers for 16E6 (F- GAGAACTGCAATGTTTCAGGACC, R- TGTATAGTTGTTTGCAGCTCTGTGC), 16E2 (F- CCATATAGACTATTGGAAACACATGCGCC, R- CTGTAGTTGCAGTTCAATTGCTTGTAATGC) (for HPV 16-Hygro Qsv) and mCherry (F- ACATCCCCGACTACTTGAAGC, R- GTAGATGAACTCGCCGTCCTG) (for mCherry Psv). The DNA was quantified based on the standard curve with plasmid DNA and converted to copy number equivalents based on the length of the plasmid.

### ECM production and infection

HaCaT cells were cultured in complete DMEM with sodium pyruvate, 10% FBS, and 1% P/S and grown to confluence by seeding 0.75–1 × 10^6^ cells onto 6 cm dishes and allowing growth for 2–3 days. Cells were washed with PBS and treated with ECM buffer containing 170 mM NH_4_OH, 0.5% Triton X-100, and PBS for 1 min. ECM buffer was removed, and ECM was gently washed 3× with PBS. Complete removal of cells was visually confirmed with light microscopy. DMEM containing sodium pyruvate, 10% FBS, and 1% P/S was added, plus either 40 µL HPV 16-Hygro Qsv (approximately 4.5 × 10^9^ copies) or 25 µL mCherry Psv (approximately 4.0 × 10^8^ copies). For uninfected control plates, DMEM without added quasi- or pseudovirus was added to the ECM. After overnight incubation, unbound Qsv/Psv was removed, media changed, and HFKs were plated at 3.0 × 10^5^ cells per plate in EpiLife media. Cells were incubated with virus for 3 days before being split to another plate with EpiLife or F Media with fibroblast feeders and 5 µg/mL hygromycin. Cells were maintained at 5–7.5 µg/mL hygromycin longitudinally.

### Monolayer differentiation of cells

For calcium differentiation of primary keratinocytes, 1.0 × 10^6^ HFKs were plated on a 10 cm tissue culture dish. After 24 h of seeding, HFKs were cultured in EpiLife medium without human keratinocyte growth supplements, with 1.8 mM calcium chloride and P/S added. Cells were trypsin harvested after 72 h or 120 h of daily calcium treatment. Untreated controls for differentiation were cells plated into a monolayer under normal EpiLife and collected at the same timepoint as differentiated samples.

For calcium differentiation of W12 cells, 1.0 × 10^6^ W12 cells were plated on a 10 cm tissue culture dish containing 3T3 fibroblast feeders, plated 24 h prior at a density of 1.0 × 10^6^. After 24 h of seeding, W12s were cultured in F-Media with the addition of 1.8 mM calcium chloride. Cells were trypsin harvested after 72 h or 120 h. Calcium treatment and F-Media were refreshed after 72 h in cells differentiated for 120 h. Untreated controls for differentiation were W12 cells plated onto feeders under normal F-Media and collected at the same timepoint as differentiated samples.

### mCherry pseudovirus infection efficiency assay

To determine the infection efficiency of mCherry Psv, HFKs were infected with mCherry Psv in EpiLife media using the ECM infection method described above. After 24, 48, and 72 h of infection, cells were imaged at 10× magnification using TXRED/*TRANS* fluorescent overlay settings on a Revolve-Discover ECHO microscope (ECHO BICO Company, San Diego, CA, US). Total fluorescence intensity relative to uninfected control images and percent red fluorescent cells across five independent fields of view were calculated using ImageJ software. Statistical significance was calculated using a two-way ANOVA with Sidak’s multiple comparisons test in GraphPad Prism version 10.6.1.

### Exonuclease V digestion and qPCR

Total genomic DNA was isolated from cells using the DNeasy Blood & Tissue Kit spin-column protocol (Qiagen, Germantown, MD, US, 69506). Briefly, cell pellets were resuspended in 200 µL PBS, and protein and RNA contaminants were removed by adding 20 µL of Proteinase K and 20 µL of RNase A (ThermoFisher Scientific, Waltham, MA, US, EN0531) and incubating for 5 min at 37°C. Cell pellets were then lysed with Buffer AL, genomic DNA isolation was performed per the manufacturer’s instructions, and DNA was eluted with 100 µL of nuclease-free water. Total DNA quantity and purity were verified using the Nanodrop Spectrophotometer (ThermoFisher Scientific, Waltham, MA, US, ND-ONEC-W). Using 2 ng of total DNA, quantitative PCR was performed to determine HPV 16 copy number. For the Exonuclease V assay, total DNA was digested using the Exonuclease V reaction. Genomic DNA of 300 ng, 1× NEB4 buffer, and 1 mM ATP were treated with or without 1 unit of ExoV (RecBCD; New England Biolabs, Ipswich, MA, US, M0345S) in a 40 µL reaction volume for 2.5 h at 37°C. Digestion was followed by heat inactivation at 95°C for 10 min. DNA was kept on ice or at −20°C until qPCR analysis. Using a 1:10 dilution of the DNA (digested and undigested), quantitative PCR was performed. All qPCR analyses were done using the QuantStudio3 Real-Time PCR System (ThermoFisher Scientific, Waltham, MA, US, A28131) with the PowerUP SYBR Green Master Mix (ThermoFisher Scientific, Waltham, MA, US, A25742). Primers for 16E6 (F- GAGAACTGCAATGTTTCAGGACC, R- TGTATAGTTGTTTGCAGCTCTGTGC) and 18S (F- GCAATTATTCCCCATGAACG, R- GGGACTTAATCAACGCAAGC) were used. Each sample was assayed in technical triplicate, and relative standard curves were generated for each target. HPV 16 DNA copy numbers were graphed relative to CaSki or W12E cell lines. Statistical significance was calculated using an unpaired, parametric, two-tailed t-test or a two-way ANOVA with Sidak’s multiple comparisons test in GraphPad Prism version 10.6.1.

### RT-qPCR

Total RNA was isolated from cell lines using the RNeasy Mini Kit (Qiagen, 74104). Briefly, cell pellets were lysed with Buffer RLT, total RNA isolation was performed per the manufacturer’s instructions, and RNA was eluted with 30 µL of RNase-free water. Total RNA quantity and purity were verified using the Nanodrop Spectrophotometer (ThermoFisher Scientific, Waltham, MA, US, ND-ONEC-W). Total RNA was used to prepare cDNA using SuperScript IV VILO with the ezDNAse enzyme kit (Invitrogen, ThermoFisher Scientific, Waltham, MA, US, 11766050) following the manufacturer’s protocol. Using a 1:45 dilution of the cDNA, quantitative RT-PCR was performed using the QuantStudio 3 Real-Time PCR System (Thermo Fisher Scientific, Waltham, MA, US, A28131) with the PowerUP SYBR Green Master Mix (Thermo Fisher Scientific, Waltham, MA, US, A25742). Primers for 16E6 (F- GAGAACTGCAATGTTTCAGGACC, R- TGTATAGTTGTTTGCAGCTCTGTGC), 16E2 (F- CCATATAGACTATTGGAAACACATGCGCC, R- CTGTAGTTGCAGTTCAATTGCTTGTAATGC), 16L1 (F- GGTGTTGAGGTAGGTCGTGG, R- CACACCTGCATTTGCTGCAT), NFX1-123 (F- CCACAGCTTCCCTCCCA, R- CCTGGACGTCAAAATAGTCAA), FLAG-tagged NFX1-123 (F- GGACTACAAAGACGACGA, R- TGCCAAGGTTGATTCTGAA), Keratin 1 (F- AGGGAGCAAATCAAGTCACTCAA, R- CTTCAGTTGGTCCACTCTCCTT), and 36B4 (F- TGCCAGTGTCTGTCTGCAGA, R- ACAAAGGCAGATGGATCAGC) were used. For Loricrin mRNA expression, amplification was carried out using TaqMan master mix and the pre-designed TaqMan probe LOR (HS01894962_s1) according to the manufacturer’s instructions. Each sample was assayed in technical triplicate, and relative standard curves were generated for each target. 36B4 was used as a reference gene for normalization of cDNA amounts. Statistical significance was calculated using an unpaired, parametric, two-tailed t-test in GraphPad Prism version 10.6.1.

### Immunoblotting

Whole-cell lysates were lysed in protein lysis buffer (50 mM Tris-HCl, pH 7.5, 150 mM NaCl, 1% NP40, 0.5% sodium deoxycholate, 0.1% SDS, 0.8% phosphatase inhibitor cocktail 2 [Sigma-Aldrich, St. Louis, MO, US, P5726], 0.8% phosphatase inhibitor cocktail 3 [Sigma-Aldrich, St. Louis, MO, US, P0044] with cOmplete Mini Protease Inhibitor Cocktail [Roche, Indianapolis, IN, US, 11836153001]). Lysates were quantified with the Pierce BCA Protein Assay (ThermoFisher Scientific, Waltham, MA, US, 23225). For differentiation studies, whole-cell lysates were prepared directly in hot 2× Laemmli sample buffer (Bio-Rad, Hercules, CA, US, 1610737) with added 2-mercaptoethanol. Lysates prepared directly in 2× sample buffer were quantified with the RC DC Protein Assay Kit (Bio-Rad, Hercules, CA, US, 5000122). A total of 25–30 µg protein lysates were loaded onto 4%–15% Mini-PROTEAN TGX precast gels (Bio-Rad, Hercules, CA, US, 4561084) and transferred to 0.2 µM PVDF membranes using the *Trans* Blot Turbo System (Bio-Rad, 12023954, 1704150). Blots were probed with antibodies: NFX1-123 at 1:5,000 (custom made by Fortis Life Sciences to peptide sequence SNLQKITKEPIIDYFDVQD), p53 (DO-1) at 1:2,500 (Cell Signaling, MA, US, 18032), p16/INK4A at 1:2,500 (Cell Signaling, Danvers, MA, US, 80772), Keratin 1 at 1:1,000 (Invitrogen, ThermoFisher Scientific, Waltham, MA, US, PA5-26699), beta-actin at 1:5,000 (Invitrogen, ThermoFisher Scientific, Waltham, MA, US, AM4302), and GAPDH at 1:300,000 (Abcam, Waltham, MA, US, ab4661). Secondary antibodies were anti-mouse HRP (1:2,500; Cell Signaling, 7076S) and anti-rabbit HRP (1:2,500; Cell Signaling, Danvers, MA, US, 7074S). Blots were visualized with SuperSignal West Pico PLUS Chemiluminescent Substrate (ThermoFisher Scientific, Waltham, MA, US, 34580) on the ChemiDoc Imaging System. Densitometry was calculated in ImageJ by measuring integrated density and normalizing to GAPDH or Beta-actin.

### Organotypic rafting

Organotypic rafting of HPV 16-Hygro Qsv-infected HFKs and W12 cells was performed as previously published (35). Rafts were fixed for 24 h at room temperature by immersion in 10% neutral buffered formalin. The specimens were then rinsed in distilled water and stored in 70% ethanol. Rafts were dehydrated through a graded series of ethanol, then cleared in xylene and infiltrated with three changes of paraffin (under vacuum at 59°C; 60 min each). Hematoxylin and eosin staining was performed using the Gemini AS automatic stainer. Images of H&E-stained rafts were taken at 20× magnification using a Revolve-Discover ECHO microscope (ECHO BICO Company, San Diego, CA, US). Raft stratification thickness was measured using ImageJ software by measuring the distance of the non-cornified stratification basal to the start of the cornified layer, as reported on the REVOLVE scope. Three fields of view of each raft were measured across five separate areas, and statistical significance was calculated by ANOVA or unpaired, parametric, two-tailed t-tests in GraphPad Prism version 10.6.1.

### Immunohistochemistry

Formalin-fixed, paraffin-embedded (FFPE) organotypic raft cultures were stained for NFX1-123 using a rabbit polyclonal anti-NFX1-123 antibody, a gift from Dr. Ann Roman, or a rabbit polyclonal anti-NFX1-123 custom made by Fortis Life Sciences (both produced to the peptide sequence SNLQKITKEPIIDYFDVQD), a mouse anti-Keratin 10 antibody (DE-K10, Invitrogen, ThermoFisher Scientific, Waltham, MA, US, MS5-13705), a mouse anti-Keratin 14 antibody (LL01, Santa Cruz Biotechnology, Santa Cruz, CA, US, sc-53253), a rabbit monoclonal anti-Loricrin antibody (Abcam, Waltham, MA, US, ab176322), a mouse polyclonal IgG control (Invitrogen, ThermoFisher Scientific, Waltham, MA, US, 31904), or a rabbit polyclonal IgG control (Invitrogen, ThermoFisher Scientific, Waltham, MA, US, 31235), each at 1 µg/mL final concentration. Antigen retrieval was done using citrate buffer pH 6.0 for 20 min at 90°C. Slides were imaged in brightfield at 20× magnification using a Revolve-Discover ECHO microscope (ECHO BICO Company, San Diego, CA, US). Semiquantitative analysis of NFX1-123 and Loricrin staining was performed using Fiji: ImageJ software. Color deconvolution for DAB was performed, and the deconvolved image displaying only the DAB channel was used for subsequent quantitation. Total intensity of a section over five independent sections was obtained. Statistical significance was calculated using an unpaired, parametric, two-tailed t-test in GraphPad Prism version 10.6.1.

### *In situ* hybridization

Briefly, FFPE organotypic raft culture specimens underwent dewaxing and a 5-min proteolysis following the Zyto*Fast* PLUS CISH Implementation Kit HRP-DAB (ZytoVision, Bremerhaven, Germany, T-1063). Specimens were hybridized overnight at 37°C using the Zyto*Fast* HPV type 16/18 probe (ZytoVision, T-1056). Post-hybridization, specimens were incubated with the provided Mouse-Anti-DIG and Anti-Mouse-HRP-Polymer as directed. Signal detection was amplified using Alexa Fluor Tyramide Conjugate 594 (Invitrogen, B40957) for 10 min at room temperature, and specimens were mounted with ProLong Diamond Antifade Mountant with DAPI (Invitrogen, P36971). Slides were imaged at 10× magnification using DAPI/TXRED fluorescent overlay settings on a Revolve-Discover ECHO microscope (ECHO BICO Company, San Diego, CA, US). The number of fluorescent puncta was calculated using a set threshold for signal in the TXRED channel using ImageJ software.

## Data Availability

All queries for data may be sent to the corresponding author.
